# Intravascular lithotripsy in coronary arteries: a review of case reports

**DOI:** 10.1186/s43044-024-00555-6

**Published:** 2024-09-07

**Authors:** Chukwuemeka A. Umeh, Harpreet Kaur, Sean Paknoosh, Benjamin Ganjian, Isha Samreen, Khabagnote Rainee, Mindy Cheng, Anisha Rastogi, Rahul Gupta

**Affiliations:** 1 Department of Internal Medicine, Hemet Global Medical Center, 1117 E. Devonshire Ave., Hemet, CA 92543 USA; 2 St. George’s University School of Medicine, West Indies, Grenada; 3 Department of Emergency Medicine, Hemet Global Medical Center, Hemet, CA USA; 4 Division of Cardiology, Hemet Global Medical Center, Hemet, CA USA; 5 Department of Internal Medicine, University of California, San Diego, CA 92122 USA

**Keywords:** Intravascular lithotripsy, Calcified coronary artery, Under-expanded stent, In-stent restenosis

## Abstract

**Background:**

Calcified coronary arteries encountered during percutaneous intervention increase the probability of unsuccessful procedures. Heavy calcification of coronary arteries may lead to suboptimal stent expansion. Intravascular lithotripsy (IVL) is a novel method of transmitting sonic waves in pulses, which fractures the calcific plaque in the vessel with minimal soft tissue injury. This study systematically reviews and summarizes the reported clinical scenarios in which IVL was successfully used in coronary lesions.

**Main text:**

Articles were obtained by searching PubMed and Embase databases for IVL use in coronary arteries. We restricted the search to case reports. Our study included 84 patients from 70 case reports/case series. The mean age was 70.3 years (SD 10) and ranged from 27 to 96 years, and 67% were males. The indications for the angiogram that led to the use of IVL include chest pain (37.7%), non-ST elevated myocardial infarction (27.9%), ST elevated myocardial infarction (13.1%), and previous under-expanded stent (8.2%). The IVL was used in the left anterior descending artery (60.7%), right coronary artery (35.7%), left main disease (23.8%), and left circumflex (9.5%). Coronary IVL was safely and successfully used in different clinical scenarios for heavily calcified coronary lesions, including in-stent restenosis of native coronary arteries, saphenous vein grafts, and under-expanded stents. In addition, IVL was successfully used synergistically with orbital and rotational atherectomy and drug-coated balloon angioplasty in select patients.

**Conclusion:**

IVL has successfully been used in an expanding array of clinical scenarios.

## Background

Calcification of coronary arteries encountered during percutaneous coronary intervention (PCI) increases the probability of an unsuccessful procedure. Heavy calcification of coronary arteries may lead to suboptimal stent expansion, interference in catheter crossing, and problems with balloon dilatation. It may also increase the risk of stent thrombosis and stent stenosis [1, 2]. There is also an increased risk of major adverse cardiovascular events (MACEs).

Recent advancements in medicine have introduced techniques to help with successful intervention in coronary arteries by providing adequate lesion preparation. Rotational atherectomy is commonly used as an intervention for severely calcified plaques. The atherectomy devices modify superficial calcium but do not modify the deep-seated calcium in a vessel, which causes a restriction in the expansion during PCI [1]. Recently, intravascular lithotripsy has been used to help defragment calcium deposition in the coronary arteries.

Intravascular lithotripsy (IVL) is a novel vessel preparation method to facilitate PCI. The technique is based on transmitting sonic waves in pulses, which fractures the calcific plaque in the vessel with minimal soft tissue injury [3]. The fracture in the calcified plaque provides improved vessel compliance and helps facilitate stent expansion. The procedure is being performed in many countries, and it has been reported to have high success rates and a low risk of complications.

There have been a growing number of reported cases of intravascular lithotripsy use in coronary and noncoronary artery vessels. Though clinicians are successfully trying out IVL in new clinical scenarios, there has not been any recent systematic review of these cases. Therefore, this study aims to systematically review and summarize the reported cases of IVL in patients with heavily calcified coronary arteries.

## Main text

Articles were obtained by searching PubMed and Embase databases with the keywords coronary artery intravascular lithotripsy. We restricted the search to case reports. Two authors independently reviewed the titles and abstracts to determine the articles that met our inclusion and exclusion criteria. To be included in the study, the article must be a case report or case series on the use of IVL in coronary arteries. We excluded articles not written in English, articles that did not include the demographic data of the cases, and conference abstracts not published in a journal. After selecting the articles that met our inclusion criteria, we reviewed the full texts and extracted the data into a spreadsheet. The data that were extracted included the age and gender of the patients, comorbidities, the coronary vessels where IVL was used, and the complications reported.

**Fig. 1:**
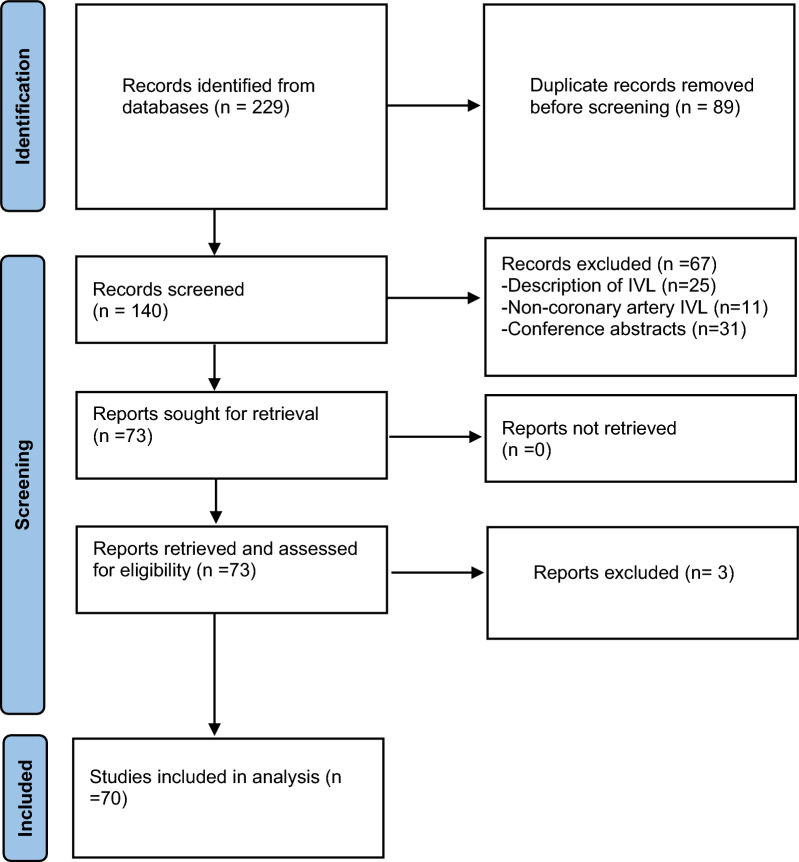
Preferred reporting items for systematic review and meta-analyses guidelines (PRISMA) flowchart of the selection process

We did a quantitative analysis using means and percentages to describe the patients' age, gender, comorbidities, and the vessels where IVL was done. We also did a qualitative analysis describing the unique situations and off-label use of IVL in calcified coronary arteries reported in the articles. We excluded articles that did not provide patient information, such as age and gender, from the quantitative analysis. However, we included such articles in the qualitative analysis (Fig. 1).

## Quantitative result

Our study included 84 patients from 70 case reports/case series. The mean age was 70.3 years (SD 10) and ranged from 27 to 96 years, and 67% were males. The major comorbidities include hypertension (35.7%), diabetes mellitus (33.3%), hyperlipidemia (22.6%), chronic kidney disease (7.1%), and chronic obstructive pulmonary disease (7.1%). The indications for the angiogram that led to the use of IVL include chest pain (37.7%), non-ST elevated myocardial infarction (27.9%), ST elevated myocardial infarction (13.1%), and previous under-expanded stent (8.2%). The IVL was used in the left anterior descending artery (60.7%), right coronary artery (35.7%), left main disease (23.8%), and circumflex artery (9.5%) (Tables 1, 2).

**Table 1:** Descriptive statistics of study participants

Age (years)	70.3 (range 27–96)	
	Number	Percentage
Gender		
Male	56	66.7%
Female	28	33.3%
Country (N = 54)		
China	2	3.7%
France	1	1.9%
India	10	18.5%
Italy	5	9.3%
Malaysia	1	1.9%
Netherland	1	1.9%
New Zealand	6	11.1%
Poland	8	14.8%
Spain	9	16.7%
Switzerland	1	1.9%
UK	3	5.6%
US	7	13.0%
Comorbidities (N = 84)		
Hypertension	30	35.7%
Diabetes	28	33.3%
Hyperlipidemia	19	22.6%
Chronic obstructive pulmonary disease	6	7.1%
Chronic kidney disease	6	7.1%
Congestive heart failure	1	1.2%
Smoker	10	11.9%
Indication for an angiogram (N = 61)		
Angina (chest pain)	23	37.7%
Non-ST elevated myocardial infarction	17	27.9%
ST elevated myocardial infarction	8	13.1%
Stent under expansion	5	8.2%
Cardiac arrest	2	3.3%
Stent restenosis	2	3.3%
Others (dyspnea, low ejection fraction, etc.)	4	6.6%
Vessels that IVL was done (N = 84)		
Left main disease	20	23.8%
Left anterior descending	51	60.7%
Left circumflex	8	9.5%
Right coronary artery	30	35.7%

**Table 2:** Description of data extracted

Paper	Age	Gender	Left main disease	LAD	LCX	RCA
Ashari, 2022 [4]	69	Male	0	1	0	0
Kaniappan, 2022 [5]	62	Female	0	1	1	0
Raza, 2022 [6]	83	Female	0	0	0	1
Xu, 2022 [7]	70	Female	0	0	0	1
Dargan, 2022 [10]	64	Male	0	1	1	0
Chu, 2022 [11]	75	Female	0	1	0	0
Priolo, 2022 [13]	62	Female	0	0	0	1
Raxwal, 2022 [14]	63	Male	0	0	0	1
Ho, 2021 [16]	81	Male	0	1	0	0
Ho, 2021 [16]	74	Female	0	1	0	0
Chiang, 2020 [21]	81	Male	0	0	0	1
Simsek, 2020 [26]	68	Female	0	1	0	0
Lee, 2021 [27]	71	Female	0	1	0	0
Donisan, 2021 [28]	27	Male	0	0	0	1
Agrawal, 2021 [29]	57	Male	1	0	0	0
Tehrani, 2020 [30]	62	Male	0	0	0	1
Górny, 2020 [31]	58	Male	0	1	0	0
Pawłowski, 2021 [32]	62	Male	0	1	0	0
Opoloski, 2019 [33]	64	Male	0	0	0	1
Yap, 2022 [34]	70	Male	0	0	0	1
Pineda, 2019 [35]	55	Male	0	1	0	0
Çimci, 2020 [36]	67	Female	1	0	0	0
Sharma, 2022 [37]	74	Male	0	0	0	1
Curtis, 2019 [38]	72	Male	0	0	0	1
Kozinski, 2020 [39]	88	Female	0	0	0	1
Tomasiewicz, 2019 [40]	72	Male	0	1	0	0
Wong, 2019 [41]	76	Male	0	0	0	1
Wong, 2019 [41]	70	Male	0	0	0	1
Wong, 2019 [41]	61	Male	0	0	1	0
Kaur, 2021 [42]	82	Male	1	1	1	0
McQuillan, 2018 [43]	61	Male	0	1	0	0
Seif, 2021 [44]	66	Male	0	1	0	0
Legutko, 2019 [45]	79	Female	0	1	0	0
Salazar, 2019 [46]	91	Female	0	1	0	0
Salazar, 2019 [46]	94	Female	0	1	0	0
Chan, 2019 [47]	74	Male	0	1	0	0
Pradhan, 2022 [48]	57	Male	0	1	0	0
Pradhan, 2022 [48]	58	Female	0	1	0	0
Ali ZA, 2020 [49]	73	Male	0	1	0	0
Marchese, 2021 [50]	78	Male	1	1	0	1
Marchese, 2021 [50]	79	Male	1	1	0	1
Dimitriadis, 2022 [51]	67	Male	0	0	0	1
Yousif, 2021 [52]	75	Male	0	1	0	0
Del Val, 2021 [53]	80	Female	1	1	1	0
Warisawa, 2019 [54]	74	Male	1	0	1	0
Tizón-Marcos, 2020 [55]	69	Female	0	1	0	0
López-Lluva, 2019 [56]	73	Female	1	1	0	0
Ciardetti, 2021 [57]	67	Male	0	0	0	1
Karacsonyi, 2021 [58]	63	Female	0	0	0	1
Hlinomaz, 2021 [59]	63	Male	0	0	0	1
Baudinet, 2021 [60]	52	Male	1	1	0	1
Rodríguez, 2019 [61]	73	Male	0	1	1	0
Rodríguez, 2019 [61]	63	Male	0	1	1	0
Rodríguez, 2019 [61]	81	Female	0	1	0	0
Azzalini, 2019 [62]	71	Female	1	1	0	0
Cicovic, 2019 [63]	73	Female	0	1	0	1
Marchese, 2020 [64]	67	Male	0	1	0	0
Marchese, 2020 [64]	73	Male	0	1	0	1
Chen, 2019 [65]	64	Male	0	0	0	1
Bawamia, 2021 [66]	86	Male	0	1	0	0
Nagaraja, 2020 [67]	67	Male	0	0	0	1
Wong, 2019 [68]	60	Female	1	1	0	0
Wong, 2019 [68]	64	Male	1	1	0	0
Wong, 2019 [68]	96	Male	1	1	0	0
Warisawa, 2020 [69]	74	Female	1	0	0	0
Sgueglia, 2019 [70]	67	Male	1	0	0	0
Jurado-Román, 2019 [71]	76	Male	0	1	0	0
Macaya, 2020 [72]	77	Male	1	1	0	0
Tovar Forero, 2020 [73]	82	Male	0	1	0	0
Morabito, 2018 [74]	77	Female	0	1	0	0
Wańczura, 2021 [75]	71	Female	0	0	0	1
Bulak, 2021 [76]	70	Male	0	0	0	1
Giacchi, 2021 [77]	67	Male	0	0	0	0
McGarvey, 2020 [78]	79	Female	0	0	0	1
Taneja, 2020 [79]	71	Female	0	0	0	1
Tumminello, 2019 [80]	68	Male	1	1	0	0
Watkins, 2019 [81]	67	Male	0	0	0	1
Ocaranza-Sánchez, 2019 [82]	83	Male	1	0	0	0
Bagur, 2022 [83]	71	Male	0	1	0	0
Bagur, 2022 [83]	70	Male	0	1	0	0
Bagur, 2022 [83]	61	Male	0	1	0	0
Bagur, 2022 [83]	70	Female	0	1	0	0
Sharma, 2022 [84]	53	Female	0	1	0	0
Kiron, 2022 [85]	63	Male	1	1	1	0
Gabryel, 2022 [86]	81	Female	0	1	0	0
Jhung Lee, 2021 [87]	71	Female	0	1	0	0
Moretti, 2021 [88]	78	Male	0	0	0	1
Goel, 2021 [89]	75	Male	1	1	0	0

## Qualitative result and discussion

### In-stent restenosis of native arteries

IVL was effectively and safely used in many in-stent restenosis cases, including in-stent restenosis secondary to under-expanded stents [4–7]. IVL has been used in calcified in-stent restenosis lesions, especially when atherectomy is technically contraindicated [4]. IVL was used in in-stent restenosis after multiple attempts with a balloon failed to expand the lesion. A drug-coated balloon angioplasty was deployed after IVL [4, 5]. Kaniappan et al. successfully deployed the same IVL balloon catheter on the left anterior descending (LAD) and left circumflex artery (LCX), showing that deploying the same IVL balloon catheter in two different vessels was feasible [5]. However, deploying the same balloon catheter on multiple vessels is now more common.

### Rotational and halfway rotational atherectomy and IVL

Rotational atherectomy is the most commonly used atherectomy approach for heavily calcified coronary artery lesions [8]. It works in a drill-like fashion with a maximum burr-to-vessel ratio of 0.7 [9]. It is recommended to treat severely calcified or fibrotic lesions that may be difficult to cross or dilate before stent placement [8]. There were reported cases of rotational atherectomy effectively and safely combined with IVL [10, 11]. In the case reported, rotational atherectomy was initially used to treat the heavily calcified lesions. Then, IVL provided further and more profound calcium modification, resulting in successful stent placement [10]. Safe and successful synergistic use of halfway rotational atherectomy with IVL was also reported [11]. In the halfway rotational atherectomy, the Burr was not advanced beyond any acute angle within the calcified lesion because of wire kinking, and IVL was then used for the lesion that the Burr did not get to [11].

### Post-dilation of under-expanded coronary stents

There were reports of IVL used in previously implanted but under-expanded coronary stents [12, 13]. Though an off-label use, IVL has been reported as safe and effective in cases where the stent was not fully expanded in the first PCI, and a second PCI was done with IVL, resulting in full stent expansion [13]. IVL was also safe and effective in treating under-expanded stents at the de novo PCI, where IVL was used for post-dilatation, resulting in full stent expansion [12]. There was also a case of a poorly expanded stent that failed treatment with several inflations with a noncompliant balloon, with persistent residual 70% stenosis. IVL was safely and successfully deployed, resulting in 0% residual stenosis [14]. Though IVL effectively treats under-expanded coronary stents, adequately modifying the plaque before stent deployment is a priority [12].

### IVL and drug-coated balloon angioplasty

Drug-coated balloon (DCB) angioplasty has emerged as an attractive strategy for leaving nothing behind during PCI [15]. DCB results in a homogenous and fast release of antiproliferative drugs into the vessel wall and inhibits neointimal hyperplasia without leaving a permanent metallic frame behind, as seen with drug-eluting stents. This eliminates the risk of in-stent thrombosis and decreases the length of dual antiplatelet therapy [16]. DCB is used for in-stent restenosis of both bare-metal and drug-eluting stents, as well as in de novo small-vessel disease and patients with high bleeding risk [17, 18].

IVL was used successfully with DCB in multiple cases. Jun Sim et al. reported seven patients safely and successfully treated with IVL and drug-coated balloon angioplasty for de novo-calcified coronary lesions [19]. Angiographic success, defined as < 30% residual stenosis, was achieved in six patients (86%), while one patient had post-procedure 50% residual stenosis. Furthermore, Ashari et al. reported that IVL was successfully used synergistically in a patient with recurrent in-stent restenosis [4]. After attempted pre-dilatation failed to achieve good lesion preparation, IVL was used. Following the use of IVL, the intravascular ultrasound showed multiple cracks within the calcified lesion. Drug-coated balloon angioplasty was then deployed, resulting in good angiographic results with good flow [4].

### IVL and orbital atherectomy

Orbital atherectomy is approved to treat severely calcified coronary artery lesions to facilitate stent delivery. Orbital atherectomy utilizes centrifugal force to create cracks in heavily calcified lesions and change the lesions' compliance and morphology [8, 20]. Chiang et al. reported a case of an unsuccessful use of IVL in a heavily calcified mid-RCA lesion. Orbital atherectomy was performed, and IVL was done again with success [21]. This case shows that IVL can be safely and effectively used synergistically with orbital atherectomy. Orbital atherectomy debulked the calcium and allowed further lesion cracking with IVL [21].

### IVL use in saphenous vein graft stenosis

Saphenous vein grafts (SVGs) are commonly used in coronary artery bypass surgery, although their long-term patency is worse than arterial bypass grafts [22]. Percutaneous coronary intervention (PCI) to SVG is sometimes done despite the high incidence of stent failure [23]. PCI to calcified saphenous vein grafts can be challenging, and the use of laser and rotational atherectomy has been reported with limited data [23]. Øksnes et al. presented a case series of five patients with calcified de novo SVG disease or SVG stent failure where IVL was successfully utilized. In four cases, IVL was used before drug-eluting stents (DES) were placed. In the fifth case, the patient had SVG in-stent restenosis, and IVL was followed by drug-eluting balloons, with good outcomes [23]. Although the use of IVL in SVG is currently off-label, these cases suggest that IVL can be safely and effectively used to treat de novo-calcified SVG lesions and SVG stent failure in selected patients.

### Success of IVL procedures

Our study showed nearly 100% clinical and angiographic success in the use of IVL in patients with heavily calcified coronary artery lesions. While the near 100% success might be due to a reporting bias because authors are more likely to publish a successful case of IVL, previous meta-analyses of IVL observational studies have reported high success rates. A systematic review and meta-analysis involving eight observational studies with 980 patients showed clinical success with IVL in 95.4% of patients and angiographic success in 97% of patients [24]. Clinical success was defined as successful stent delivery with IVL that results in less than 50% residual diameter stenosis without in-hospital major adverse cardiac events (MACEs), such as myocardial infarction, or revascularization of the same lesion after the completion of the initial procedure. Angiographic success was defined as successful stent delivery with IVL that results in less than 50% residual diameter stenosis without significant angiographic complications such as coronary perforation, persistent slow flow, no-reflow, or abrupt closure [24]. Similarly, multiple systematic reviews of IVL have reported significant improvement in the post-IVL lumen diameter and a significant reduction in the luminal calcium angle and maximum calcium thickness [2, 24, 25].

## Limitations of the study

One limitation of our review of case reports is that the study has a small sample size. Small sample sizes can lead to variability in outcomes and may not fully capture the diversity of patient responses to IVL. Furthermore, authors will likely publish cases that had a successful outcome, leading to publication bias. This potential publication bias could skew the overall impression of IVL's efficacy and safety. The high success rates reported may not accurately reflect the real-world performance of IVL, where less favorable outcomes might be underreported. Therefore, more studies are required to assess the efficacy and safety of IVL in some of the clinical scenarios described in the articles. Additionally, this review focuses on short-term procedural success and immediate angiographic results, as the case reports do not address long-term outcomes, including the results' durability and potential late complications. Finally, given the small number of cases and the potential for selective reporting, the generalizability of the findings to broader patient populations is limited.

## Conclusions

Our study showed that IVL has been successfully used in different clinical scenarios for heavily calcified coronary lesions, including in-stent restenosis of native coronary arteries, SVG, and under-expanded stents. In addition, IVL was successfully used synergistically with orbital and rotational atherectomy and drug-coated balloon angioplasty in select patients.

## Data Availability

All papers analyzed during this study are included in this published article in Table 2.

## Abbreviations

DCB: Drug-coated balloon
DES: Drug-eluting stents
IVL: Intravascular lithotripsy
LAD: Left anterior descending artery
LCX: Left circumflex artery
MACE: Major adverse cardiovascular events
PCI: Percutaneous coronary intervention
RCA: Right coronary artery
SVG: Saphenous vein grafts
